# Orlistat: a comprehensive review of a lipase inhibitor from obesity management to emerging oncological applications

**DOI:** 10.3389/fphar.2026.1944242

**Published:** 2026-09-15

**Authors:** Li Li, Yuchao Hao

**Affiliations:** 1 Department of Clinical Nutrition, The Second Hospital of Dalian Medical University, Dalian, China; 2 Department of Hematology, The Second Hospital of Dalian Medical University, Dalian, China

**Keywords:** anticancer therapy, cancer metabolism, drug repurposing, fatty acid synthase (FASN), lipid metabolism, obesity, Orlistat

## Abstract

Orlistat, a potent inhibitor of gastric and pancreatic lipases, has been a cornerstone of pharmacological obesity management for over 2 decades. By reducing dietary fat absorption, it facilitates modest but clinically significant weight loss and helps prevent weight regain when combined with lifestyle interventions. Its use is associated with improvements in a range of obesity-related comorbidities, including enhanced glycemic control, reduced risk of type 2 diabetes progression, and favorable modifications of cardiovascular risk factors such as dyslipidemia. However, its clinical utility is often limited by predictable gastrointestinal adverse effects, which can impact patient adherence. Beyond its established role in weight control, a growing body of preclinical evidence has unveiled Orlistat’s off-target activity as an inhibitor of fatty acid synthase (FASN), a key enzyme in *de novo* lipogenesis that is frequently overexpressed in various cancers. This discovery has spurred investigation into its potential as an anti-neoplastic agent, with studies demonstrating its ability to induce apoptosis, arrest cell cycle progression, and synergize with immunotherapy in cancer models. This review provides a comprehensive analysis of Orlistat, covering its molecular mechanisms, clinical efficacy and safety in diverse populations, impact on metabolic health, and the exciting, yet nascent, evidence for its repurposing in oncology.

## 1 Introduction

The escalating global prevalence of obesity represents one of the most significant public health challenges of the 21st century. This complex, multifactorial chronic disease is characterized by excessive adiposity that poses a substantial risk to individual health and places an immense burden on healthcare systems worldwide. The clinical consequences of obesity are profound, as it is a primary driver for a cluster of metabolic comorbidities, including type 2 diabetes (T2D), cardiovascular diseases (CVD) such as hypertension and dyslipidemia, and metabolic dysfunction-associated steatotic liver disease (MASLD), formerly known as nonalcoholic fatty liver disease (NAFLD), which can progress to cirrhosis and hepatocellular carcinoma. While lifestyle interventions focusing on diet and physical activity remain the cornerstone of weight management, their long-term efficacy is often limited, necessitating adjunctive therapeutic strategies for sustained weight loss and comorbidity risk reduction.

The history of anti-obesity pharmacotherapy has been fraught with challenges, with numerous agents withdrawn from the market due to safety concerns, particularly cardiovascular and psychiatric adverse events. This has left a significant therapeutic gap, with only a few medications approved for long-term use. Among these, Orlistat (tetrahydrolipstatin), a potent and specific inhibitor of gastrointestinal lipases, has maintained a consistent presence in the clinical armamentarium since its approval in the late 1990s. Originally isolated from *Streptomyces toxytricini*, Orlistat acts locally in the gut to prevent the hydrolysis of dietary triglycerides into absorbable free fatty acids and monoglycerides, thereby reducing caloric intake from fat by approximately 30%. Its efficacy has been established in numerous large-scale, long-term clinical trials demonstrating modest but significant weight loss, prevention of weight regain, and improvement in cardiovascular risk factors over periods of up to 4 years when used as an adjunct to a hypocaloric diet (Sjostrom et al., 1998; Davidson et al., 1999). The utility of Orlistat has been demonstrated not only for initial weight loss but also for the critical phase of weight maintenance following a very-low-energy diet (Richelsen et al., 2007). Furthermore, its application has been extended and studied in specific populations, including obese adolescents, where it has shown efficacy in improving weight and body composition (Chanoine et al., 2005).

This review aims to synthesize the extensive body of literature on Orlistat, providing a comprehensive overview of its established and emerging roles in medicine. We will first examine its primary mechanism of action as a lipase inhibitor and its downstream physiological effects, such as impacts on gastric emptying and insulin secretion (Borovicka et al., 2000; Damci et al., 2004). Subsequently, we will critically appraise the clinical evidence supporting its efficacy for weight management and its significant impact on obesity-related comorbidities. This includes a detailed discussion of its role in improving glycemic control in patients with T2D, both with and without concomitant therapies like metformin or insulin (Hollander et al., 1998; Kelley et al., 2002; Miles et al., 2002), its landmark contribution to the prevention of T2D in high-risk individuals as demonstrated in the XENDOS study (Torgerson et al., 2004), and its beneficial effects on cardiovascular risk profiles, including endothelial function (Bergholm et al., 2003). The safety and tolerability profile of Orlistat will be thoroughly reviewed, addressing its well-known gastrointestinal side effects and examining the evidence regarding rarer but more severe concerns, such as acute liver injury (Douglas et al., 2013). Finally, this review will explore the paradigm-shifting discovery of Orlistat’s off-target effects, particularly its potent inhibition of fatty acid synthase (FASN), a key enzyme in *de novo* lipogenesis that is highly overexpressed in many human cancers (Kridel et al., 2004; Pemble et al., 2007). We will delve into the preclinical evidence supporting its potential as an anti-cancer agent, with demonstrated pro-apoptotic activity in malignancies such as chronic lymphocytic leukemia (Pallasch et al., 2008), as elucidated through activity-based proteome profiling (Yang et al., 2010). This discussion will be balanced by an evaluation of large-scale epidemiological data concerning cancer risk in Orlistat users, such as the finding of no increased risk for colorectal cancer (Hong et al., 2013), thereby providing a multifaceted perspective on the evolving therapeutic landscape of this established anti-obesity drug.

### 1.1 Literature search strategy and evidence appraisal

This review was designed as a narrative review to provide an integrated and critical overview of the pharmacological properties, clinical applications, safety profile, and emerging oncological potential of Orlistat. A literature search was conducted using PubMed/MEDLINE, with coverage from database inception to August 2026. The search strategy combined terms related to Orlistat and its major pharmacological and therapeutic contexts, including “Orlistat,” “lipase inhibition,” “fatty acid synthase,” “FASN,” “obesity,” “type 2 diabetes,” “cardiovascular disease,” “cancer,” “oncology,” “lipid metabolism,” and “drug repurposing.” Reference lists of relevant reviews and key primary studies were also examined to identify additional publications of clinical or mechanistic relevance.

Priority was given to randomized controlled trials, systematic reviews and meta-analyses, large observational studies, and well-characterized preclinical studies that provided substantial clinical, pharmacological, or mechanistic evidence. Studies were considered for inclusion when they addressed the efficacy, safety, pharmacological mechanisms, metabolic effects, or potential oncological applications of Orlistat. Studies with limited relevance to the central scope of this review, publications lacking sufficient methodological information, and reports focused exclusively on unrelated lipase inhibitors or lipid-metabolism pathways were excluded.

Because the evidence base for Orlistat differs substantially between metabolic medicine and oncology, evidence was interpreted according to study type and translational maturity. Clinical efficacy and safety conclusions were primarily based on randomized controlled trials, systematic reviews, meta-analyses, and large observational studies, whereas mechanistic and oncological claims were evaluated in the context of their predominantly preclinical evidence base. In particular, findings from cell-based and animal studies were not considered equivalent to clinical evidence and are presented as hypothesis-generating or translationally promising rather than as evidence of established therapeutic efficacy in humans. Particular attention was paid to study design, population size, clinical relevance, consistency across studies, and the limitations of extrapolating preclinical findings to clinical practice.

## 2 Mechanisms of action of Orlistat: primary lipase inhibition and secondary systemic effects

### 2.1 Primary mechanism: inhibition of gastrointestinal lipases

Orlistat, a hydrogenated derivative of the natural product lipstatin, exerts its primary therapeutic effect through the potent, specific, and irreversible inhibition of gastrointestinal lipases. Its mechanism is centered on the covalent modification of the active site of these enzymes, primarily gastric and pancreatic lipases, which are crucial for the digestion of dietary fats (Bello et al., 2017). Structurally, Orlistat mimics a triglyceride substrate, allowing it to enter the active site of the lipase. Here, the strained β-lactone ring of Orlistat is susceptible to nucleophilic attack by the catalytic serine residue (e.g., Ser152 in human pancreatic lipase). This reaction forms a stable, covalent ester bond between the drug and the enzyme, rendering the lipase inactive (Bello et al., 2017) (Figure 1).

**FIGURE 1:**
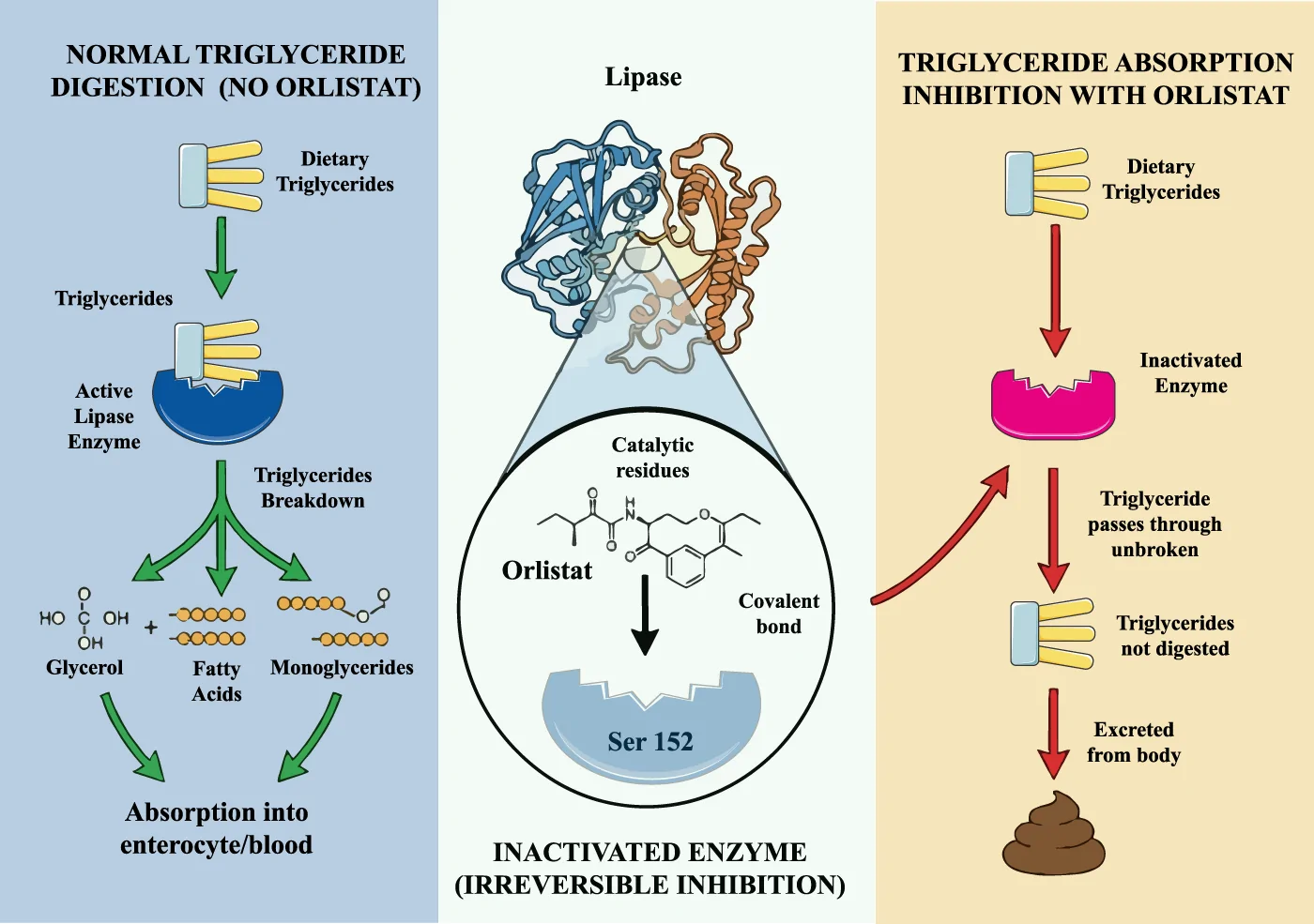
Schematic diagram of Orlistat acting on lipase. Orlistat binds covalently to the active site serine (Ser152), blocking triglyceride hydrolysis, reducing fat absorption (∼30%) and increasing fecal fat excretion.

By inactivating these key digestive enzymes, Orlistat effectively prevents the hydrolysis of dietary triglycerides into absorbable free fatty acids and monoglycerides. This targeted inhibition within the gastrointestinal lumen leads to a significant reduction in the absorption of dietary fat by approximately 30% (Davidson et al., 1999; Bello et al., 2017). The unabsorbed triglycerides are subsequently excreted in the feces, resulting in a caloric deficit that contributes to weight loss.

A direct physiological consequence of this mechanism is the attenuation of postprandial lipemia, characterized by a blunted rise in plasma triglyceride levels following a high-fat meal (Reitsma et al., 1994). The presence of undigested fat in the gastrointestinal tract also modulates gut physiology; studies have shown that inhibiting fat digestion with Orlistat alters gastrointestinal sensations, such as fullness and nausea, that are normally induced by duodenal lipids (Feinle et al., 2001). Furthermore, the induced fat malabsorption can have other metabolic consequences. The unabsorbed fatty acids can form insoluble calcium soaps in the intestinal lumen, a process that reduces the amount of free calcium available to bind with dietary oxalate. This consequently increases the intestinal absorption of free oxalate, leading to a significant rise in urinary oxalate excretion and a potential risk for hyperoxaluria and oxalate nephropathy in susceptible individuals (Ferraz et al., 2004).

### 2.2 Secondary (off-target) mechanisms

Beyond its well-defined role in the gut, Orlistat exhibits several secondary or “off-target” pharmacological actions that are gaining recognition for their therapeutic potential, particularly in oncology and metabolic regulation. These systemic effects arise from the absorption of a small fraction of the drug and its interaction with intracellular enzymes that share structural homology with gastrointestinal lipases.

The most extensively studied off-target effect is the potent inhibition of the intracellular enzyme FASN. FASN is a critical enzyme responsible for the *de novo* synthesis of palmitate and is highly overexpressed in many human cancers, where it provides lipids for membrane biogenesis, energy storage, and signaling molecules essential for rapid proliferation and survival (Kridel et al., 2004). Orlistat targets the thioesterase (TE) domain of FASN, which catalyzes the final step of fatty acid synthesis. Similar to its action on lipases, Orlistat forms a covalent adduct with the catalytic serine residue of the TE domain, effectively blocking its function. Structural studies, including X-ray crystallographic analyses of Orlistat-associated lipase complexes and related serine hydrolase models, have provided structural evidence supporting the covalent interaction between the β-lactone ring of orlistat and the catalytic serine residue (Pemble et al., 2007; Fako et al., 2014; Kitadokoro et al., 2020). The inhibition of FASN by Orlistat disrupts phospholipid synthesis, leading to profound cellular stress. This has been shown to induce robust endoplasmic reticulum (ER) stress and subsequent apoptosis in tumor cells, highlighting a key mechanism for its antineoplastic activity (Little et al., 2007).

Orlistat’s inhibitory activity extends to other lipases and serine hydrolases. For instance, it has been shown to inhibit lipoprotein lipase (LPL) in B-cell chronic lymphocytic leukemia (CLL) cells, an effect linked to the induction of apoptosis in these malignant cells (Pallasch et al., 2008). While direct inhibition is less characterized for other lipases, the role of enzymes like endothelial lipase (LIPG) in supplying lipid precursors for breast cancer growth (Slebe et al., 2016) and the monoacylglycerol lipase α/β-Hydrolase Domain-6 (ABHD6) in controlling lipid signaling for insulin secretion (Zhao et al., 2014) underscores the therapeutic potential of targeting lipid metabolic pathways that may be susceptible to Orlistat or its analogues.

Finally, Orlistat indirectly modulates gut hormone signaling. By preventing the digestion and absorption of fats in the upper small intestine, it increases the delivery of lipids to the distal ileum and colon. This stimulates the enteroendocrine L-cells in this region to enhance the secretion of glucagon-like peptide-1 (GLP-1) (Damci et al., 2004). This augmentation of postprandial GLP-1 is clinically significant, as GLP-1 is an incretin hormone that improves glycemic control by stimulating glucose-dependent insulin secretion and suppressing glucagon release. This effect may contribute to the improvements in insulin resistance and metabolic control observed in patients with type 2 diabetes treated with Orlistat, independent of weight loss alone (Damci et al., 2004; Kelley et al., 2004).

## 3 Clinical efficacy in weight management

Orlistat is one of the longest-standing pharmacotherapies approved for weight management, and its efficacy has been established through numerous randomized controlled trials (RCTs) and meta-analyses (Table 1). When used as an adjunct to a hypocaloric diet and lifestyle modification, Orlistat consistently produces modest but statistically significant weight loss compared to placebo. Landmark long-term studies, such as the 2-year European Multicentre Orlistat Study Group trial and a parallel US-based study, demonstrated that patients treated with Orlistat (120 mg three times daily) achieved greater weight loss than those on placebo plus diet alone. In the first year, the Orlistat groups lost approximately 8.8%–10.2% of their initial body weight, compared to 5.8%–6.1% in the placebo groups, resulting in a placebo-subtracted weight loss of around 3% (Sjostrom et al., 1998; Davidson et al., 1999).

**TABLE 1:** Clinical efficacy of Orlistat in weight management.

Study	Population	Dose	Duration	Weight loss	References
European multicentre Orlistat Study Group	Obese adults	120 mg TID	2 years	10.2%	Sjostrom et al. (1998)
US-based RCT	Obese adults	120 mg TID	2 years	8.8% (8.76 kg)	Davidson et al. (1999)
XENDOS study	Obese patients with impaired glucose tolerance	120 mg TID	4 years	5.3% (5.8 kg)	Torgerson et al. (2004)
Obese adolescents RCT	Obese adolescents (12–16 years)	120 mg TID	54 weeks	BMI −0.55 kg/m^2^ vs. + 0.31 placebo	Chanoine et al. (2005)
Type 2 diabetes RCT	Obese patients with type 2 diabetes	Various	6 months	10.2% (10.1 kg)	Kelley et al. (2004)
Network meta-analysis	Obese women with polycystic ovary syndrome (PCOS)	Various	Multiple trials	2.6%	Wang et al. (2018)

A comprehensive meta-analysis of long-term (≥1 year) RCTs confirmed these findings, reporting a mean additional weight loss of 2.9 kg (95% CI 2.5–3.2) for Orlistat users compared to placebo (Rucker et al., 2007). Another meta-analysis similarly found a placebo-subtracted weight reduction of 2.89 kg (95% CI 2.27–3.51) (Li et al., 2005). This degree of weight loss, although modest, is associated with significant improvements in cardiometabolic risk factors, including blood pressure, lipid profiles, and glycemic control (Rucker et al., 2007). Indeed, health economic modeling based on clinical trial data suggests that the weight loss and improved cardiovascular risk profile achieved with Orlistat in obese patients with type 2 diabetes translate into long-term reductions in microvascular and macrovascular complications (Lamotte et al., 2002).

A critical aspect of obesity management is the prevention of weight regain. Long-term data show that Orlistat aids in the sustainability of weight loss. In 2-year trials, participants continuing Orlistat treatment experienced significantly less weight regain in the second year compared to those on placebo (Sjostrom et al., 1998; Davidson et al., 1999). A systematic review focusing on long-term weight loss maintenance found that pharmacological interventions, including Orlistat, combined with lifestyle support, are more effective for preventing weight regain than lifestyle interventions alone (Dombrowski et al., 2014). Perhaps the most compelling evidence for Orlistat’s long-term benefit comes from the XENical in the Prevention of Diabetes in Obese Subjects (XENDOS) study, a 4-year trial which demonstrated that Orlistat, as an adjunct to lifestyle changes, significantly reduced the cumulative incidence of type 2 diabetes in obese individuals with impaired glucose tolerance (Torgerson et al., 2004). Systematic reviews and meta-analyses have quantified this effect, showing that Orlistat reduces the risk of progressing to diabetes by approximately 37% (RR 0.63, 95% CI 0.46–0.86) (Padwal et al., 2005; Gillies et al., 2007).

The efficacy of Orlistat has also been evaluated in several special populations. In obese adolescents, a 54-week RCT found that Orlistat plus behavioral therapy resulted in a significantly greater reduction in body mass index (BMI) (−0.55 kg/m^2^) compared to placebo plus therapy (+0.31 kg/m^2^) (Chanoine et al., 2005). Meta-analyses of trials in this population confirm that Orlistat produces a modest but significant reduction in BMI and may improve lipid profiles and blood pressure (Czernichow et al., 2010). Evidence reviews for the US Preventive Services Task Force have also supported its use as a treatment option for adolescent obesity (O’Connor et al., 2017). In women with polycystic ovary syndrome (PCOS), a condition strongly associated with obesity and insulin resistance, Orlistat has been shown to be effective for weight loss. A systematic review and network meta-analysis found that Orlistat was superior to metformin for weight reduction in this population, although less effective than GLP-1 receptor agonists (Goldberg et al., 2024). Furthermore, Orlistat has shown utility in patients with binge eating disorder (BED); a randomized trial found that adding Orlistat to guided self-help cognitive behavioral therapy resulted in significantly greater weight loss and a larger reduction in binge frequency compared to placebo (Grilo et al., 2005).

As an adjunct to lifestyle and behavioral interventions, Orlistat fits well within the primary care setting, where most obesity management is initiated. Evidence reviews of primary care-relevant treatments have shown that pharmacotherapy combined with behavioral support results in greater weight loss than behavioral interventions alone (Leblanc et al., 2011). However, in the current landscape of anti-obesity medications (AOMs), the relative efficacy of Orlistat is modest (Table 2). Numerous network meta-analyses have consistently demonstrated that newer agents provide superior weight loss. One analysis reported a placebo-subtracted weight loss of 2.6% for Orlistat, compared to 5.3% for liraglutide 3.0 mg, 6.8% for naltrexone-bupropion, and 8.8% for phentermine-topiramate (Khera et al., 2016). More recent, comprehensive network meta-analyses that include newer GLP-1 receptor agonists have further highlighted this disparity, showing that agents like semaglutide 2.4 mg can achieve placebo-subtracted weight loss exceeding 12%, far surpassing the ∼3% achieved with Orlistat (Shi et al., 2024). Head-to-head data, though limited, support these findings; a randomized trial with an open-label Orlistat arm found that liraglutide produced significantly greater weight loss (Astrup et al., 2009). Consequently, recent clinical practice guidelines, such as those from the American Gastroenterological Association, recommend newer, more effective AOMs over Orlistat for most patients, though Orlistat remains a viable option, particularly given its different mechanism of action and safety profile (Grunvald et al., 2022).

**TABLE 2:** Comparative characteristics of orlistat and contemporary anti-obesity pharmacotherapies.

Feature	Orlistat	GLP-1 receptor agonists	Newer incretin-based therapies
Primary target	Gastric/pancreatic lipases	GLP-1 receptor	Incretin pathways
Major site of action	Gastrointestinal tract	Systemic/peripheral	Systemic/peripheral
Administration	Oral	Oral or injectable, agent-dependent	Agent-dependent
Weight-loss efficacy	Modest	High	Very high in some agents
Systemic exposure	Limited	Substantial	Substantial
GI adverse effects	Common	Common	Common
Long-term clinical experience	Extensive	Increasing	More limited
Major limitation	GI tolerability; vitamin malabsorption	GI tolerability; cost/access	Cost/access; long-term evidence evolving

Therefore, the therapeutic position of Orlistat should be considered in the context of contemporary anti-obesity therapies. Compared with GLP-1 receptor agonists, Orlistat generally produces more modest weight loss, although its predominantly gastrointestinal action, limited systemic exposure, oral administration, and extensive clinical experience distinguish it from newer systemic therapies. Thus, while GLP-1 receptor agonists may be preferred when substantial weight reduction is the primary goal, Orlistat may remain an option for selected patients based on individual clinical characteristics, tolerability, accessibility, and treatment preferences.

## 4 Impact on obesity-related comorbidities

The therapeutic benefits of Orlistat extend beyond weight reduction to significantly ameliorate a spectrum of obesity-related comorbidities. This section details the impact of Orlistat-induced weight loss on major metabolic and cardiovascular outcomes, including T2D, dyslipidemia, hypertension, MASLD, CKD, and heart failure (Table 3).

**TABLE 3:** Effects on obesity-related comorbidities.

Condition	Study	Population	Dose	Main outcome	References
T2D Prevention	XENDOS study	Obese adults	120 mg TID	Reduced cumulative T2D incidence by 37%	Torgerson et al. (2004), Padwal et al. (2005), Gillies et al. (2007)
Glycemic Control	Hollander et al. (1998)	Obese adults	120 mg TID	Decreases in HbA1c and fasting plasma glucose, improved insulin sensitivity	Hollander et al. (1998)
Dyslipidemia	Meta-regression analyses	Obese adults	Various	Slightly reduces cholesterol and triglyceride levels, but not lipoprotein(a) levels	Sahebkar et al. (2017)
Endothelial Function	Bergholm et al. (2003)	Obese premenopausal nondiabetic women with a history of gestational diabetes	120 mg TID	LDL reduction improved vasodilation	Bergholm et al. (2003)
MASLD/MASH	Harrison et al. (2009)	Overweight with MASH	120 mg TID	Improves serum ALT levels and steatosis	Harrison (2003), Zelber-Sagi et al. (2006), Harrison et al. (2009)
CKD	MacLaughlin et al. (2010)	Obese adults with CKD and diabetes, hypertension, or dyslipidemia and otherwise suitable for kidney transplant if on dialysis therapy	120 mg TID	Weight loss, renal function stabilization	MacLaughlin et al. (2010)
Heart Failure	Systematic review	Obese adults with heart failure	Various	Functional improvement	McDowell et al. (2018), Peck et al. (2024)

### 4.1 Prevention of type 2 diabetes

The landmark XENDOS (XENical in the prevention of Diabetes in Obese Subjects) study provided pivotal evidence for Orlistat’s role in diabetes prevention (Torgerson et al., 2004). This 4-year, randomized, double-blind trial in obese patients with normal or impaired glucose tolerance demonstrated that Orlistat (120 mg three times daily) combined with lifestyle changes reduced the cumulative incidence of T2D by 37.3% compared to lifestyle changes alone. This risk reduction was attributed to greater weight loss and improved insulin sensitivity in the Orlistat group. This finding is supported by systematic reviews confirming that pharmacological interventions, including Orlistat, can delay or prevent T2D in high-risk populations (Padwal et al., 2005; Gillies et al., 2007).

### 4.2 Glycemic control and insulin sensitivity in established type 2 diabetes

In patients with established T2D, Orlistat adjunctive therapy confers significant metabolic benefits. A 1-year randomized, double-blind study demonstrated that Orlistat plus diet led to greater weight loss and more pronounced improvements in glycemic control (HbA1c and fasting plasma glucose) and insulin sensitivity compared to placebo in obese patients with T2D (Hollander et al., 1998). A subsequent systematic review and meta-analysis of randomized controlled trials confirmed that Orlistat improves glycemic control in overweight and obese patients with T2D, with effects partly independent of weight loss (Aldekhail et al., 2015). However, whether these metabolic benefits are attributable to direct pharmacological effects of Orlistat or primarily reflect weight loss remains difficult to determine.

The drug’s mechanism may involve modulating postprandial metabolism; lipase inhibition with Orlistat has been shown to augment the postprandial increase in glucagon-like peptide 1 (GLP-1), an incretin hormone that stimulates insulin secretion, in obese patients with T2D (Damci et al., 2004). Furthermore, Orlistat improves insulin resistance and regional adiposity in type 2 diabetes, as evidenced by studies examining weight loss-dependent and -independent effects (Kelley et al., 2004). Overall, the clinical evidence supporting Orlistat is substantially stronger than that supporting its oncological repurposing, but the magnitude of weight loss is modest compared with newer incretin-based therapies.

### 4.3 Improvement in cardiovascular risk factors

Orlistat therapy positively impacts the cardiovascular risk profile through multiple pathways. A systematic review and meta-analysis of 33 randomized controlled trials concluded that Orlistat significantly reduces plasma total cholesterol, low-density lipoprotein (LDL) cholesterol, and triglycerides, with the lipid-lowering effects being partly independent of weight loss (Sahebkar et al., 2017). Network meta-analyses of weight-loss medications rank Orlistat favorably for its beneficial effects on cardiometabolic risk profiles, including lipid parameters (Khera et al., 2018). Beyond lipids, Orlistat-induced weight loss contributes to blood pressure reduction. Long-term studies in obese patients at high coronary risk show that Orlistat improves overall coronary heart disease risk profiles (Lindgarde, 2000). Importantly, the cardiovascular benefits may involve direct vascular effects. In obese women with previous gestational diabetes, Orlistat-induced lowering of LDL cholesterol, rather than moderate weight loss *per se*, was associated with improved endothelium-dependent vasodilatation, a key marker of vascular health (Bergholm et al., 2003). Long-term weight loss with Orlistat also reduces non-traditional cardiovascular risk factors such as interleukin-18 and matrix metalloproteinase-9 (Madsen et al., 2009). Real-world evidence suggests these physiological benefits translate into clinical practice, as initiation of Orlistat is associated with a subsequent decrease in the use of cardiovascular medications (Czernichow et al., 2012). However, improvements in surrogate cardiovascular risk factors should not be interpreted as definitive evidence of reductions in cardiovascular morbidity or mortality. Meanwhile, improvements in cardiovascular risk factors should not be equated with definitive reductions in cardiovascular events.

### 4.4 Management of metabolic dysfunction-associated steatotic liver disease (MASLD) and metabolic dysfunction-associated steatohepatitis (MASH)

MASLD, often a hepatic manifestation of metabolic syndrome, benefits from Orlistat-induced weight loss. A randomized, prospective trial in overweight subjects with biopsy-proven MASH found that 36 weeks of treatment with Orlistat plus caloric restriction resulted in significant weight loss and improved liver histology (including reductions in steatosis, inflammation, and fibrosis) compared to diet alone (Harrison et al., 2009). An earlier double-blind, randomized, placebo-controlled trial also reported that Orlistat treatment for 6 months in patients with MASLD led to significant reductions in liver enzymes and improved ultrasound findings of hepatic steatosis (Zelber-Sagi et al., 2006). Case series further support the utility of Orlistat in MASH management, linking weight reduction to biochemical and histological improvement (Harrison and S., 2003). These findings align with broader systematic reviews indicating that weight reduction is a cornerstone of effective MASLD treatment (Peng et al., 2011). However, the extent to which these hepatic benefits are independent of weight loss remains uncertain.

### 4.5 Effects in chronic kidney disease (CKD) and heart failure

Obesity is a significant risk factor for CKD progression and complicates the management of heart failure. In obese patients with CKD, a nonrandomized trial of a structured program involving Orlistat, nutrition education, diet, and exercise demonstrated successful weight loss and stabilization of renal function over a 2-year follow-up period, suggesting a potential role in slowing CKD progression (MacLaughlin et al., 2010). Regarding heart failure, a systematic review of intentional weight loss strategies (including pharmacotherapy with agents like Orlistat) in patients with obesity and heart failure found that interventions were generally associated with improvements in functional status, quality of life, and left ventricular ejection fraction, though evidence on hard outcomes like mortality remains limited (McDowell et al., 2018). A more recent systematic review specifically noted the scarcity of data from pharmacotherapy trials in this population but indicated trends toward symptomatic benefit (Peck et al., 2024).

In summary, Orlistat-induced weight loss delivers multifaceted therapeutic benefits that mitigate the burden of key obesity-related comorbidities. By preventing T2D, improving glycemic control and insulin sensitivity, ameliorating dyslipidemia and hypertension, enhancing endothelial function, reducing liver fat and inflammation in MASLD/MASH, and potentially stabilizing renal function and improving heart failure symptoms, Orlistat addresses the core pathophysiological links between excess adiposity and cardiometabolic disease (Table 4).

**TABLE 4:** Evidence level, therapeutic effects, and translational limitations of Orlistat.

Therapeutic area	Evidence type	Main evidence	Evidence strength	Major limitation
Weight management	RCTs/meta-analyses	Modest weight loss	High	Limited magnitude of weight loss
T2D prevention	RCT/meta-analysis	Reduced diabetes incidence	High	Benefit partly related to weight loss
Dyslipidemia/CVD risk	RCTs/meta-analyses	Improved lipid parameters	Moderate–high	Limited evidence for hard CV outcomes
MASLD/MASH	RCTs/small clinical studies	Improvements in metabolic/liver parameters	Moderate	Limited histological and long-term evidence
Cancer	Cell/animal studies	FASN inhibition, apoptosis, metabolic disruption	Low/preclinical	Lack of clinical validation
Cancer combinations	Cell/animal studies	Potential synergy with immunotherapy/endocrine therapy	Low/preclinical	Dose, PK, and translational uncertainty

## 5 Safety, tolerability, and adverse events

The safety and tolerability profile of Orlistat is well-characterized and is dominated by adverse events that are a direct pharmacological consequence of its mechanism of action. While generally considered safe for long-term use, its tolerability, particularly concerning gastrointestinal side effects, is a primary determinant of patient adherence and therapeutic success (Table 5).

**TABLE 5:** Safety and adverse events.

Adverse event	Incidence	Mechanism/Notes	References
GI: oily spotting, steatorrhea, flatus	High	Direct effect of fat malabsorption	Johansson et al. (2009), Schroll et al. (2016)
Acute liver injury	Rare	Not confirmed in large cohort	Douglas et al. (2013)
Acute oxalate nephropathy	Rare	Fat malabsorption secondary to acute oxalate nephropathy	Ferraz et al. (2004), Singh et al. (2007)
Colorectal cancer	Not increased	Large matched cohort study	Hong et al. (2013)
Fat-soluble vitamin deficiency	Moderate	Inhibiting fat digestion	Itoe et al. (2024)
Cyclosporine interaction	Rare	Reduced intestinal absorption	Evans et al. (2003)

### 5.1 Gastrointestinal adverse events: mechanism, incidence, and management

The most frequently reported adverse events associated with Orlistat are gastrointestinal (GI) in nature and stem directly from its mode of action: the inhibition of gastric and pancreatic lipases. By preventing the hydrolysis of dietary triglycerides into absorbable free fatty acids and monoglycerides, Orlistat increases the concentration of unabsorbed fat in the lumen of the lower intestine. This leads to a predictable constellation of symptoms, including oily spotting from the rectum, flatus with discharge, fecal urgency, steatorrhea (oily or fatty stools), and increased defecation (Johansson et al., 2009). The incidence of these events is high, particularly during the initial phase of treatment and following the consumption of high-fat meals. These predictable, albeit often bothersome, side effects are a leading cause of treatment discontinuation, with meta-analyses showing significantly higher rates of withdrawal due to adverse events for Orlistat compared to placebo (Fabricatore et al., 2009; Johansson et al., 2009).

A critical analysis of how these adverse events are reported reveals potential discrepancies between internal clinical study reports (CSRs) and final published papers. A detailed document analysis found that CSRs often contain more comprehensive data on adverse events, suggesting that published literature may sometimes underrepresent the full burden of these side effects on patients (Schroll et al., 2016). The management of these GI events is centered on patient education and dietary modification. Adherence to a mildly hypocaloric, low-fat diet (with approximately 30% of calories from fat) significantly mitigates the severity and frequency of these symptoms. In this way, the GI side effects can act as a form of negative behavioral reinforcement, discouraging high-fat dietary choices (Schroll et al., 2016).

Beyond these common side effects, the active form of Orlistat, tetrahydrolipstatin (THL) (Mulzer et al., 2014), has been investigated preclinically for its effects in other GI pathologies. For instance, in experimental models of acute pancreatitis in rats, where lipolytic enzymes are implicated in pathogenesis, the lipase inhibitor THL was studied as a potential therapeutic modality, highlighting the complex role of lipolysis in GI physiology and pathology (Kimura et al., 1992; Noel et al., 2016).

### 5.2 Systemic safety and rare adverse events

While Orlistat has minimal systemic absorption, several rare but serious systemic adverse events have been evaluated in post-marketing surveillance and large-scale studies.

#### 5.2.1 Acute liver injury

Post-marketing case reports of severe liver injury raised concerns about Orlistat’s hepatotoxicity. However, a large, population-based self-controlled case series study using the UK Clinical Practice Research Datalink, which included nearly 95,000 Orlistat users, found no evidence of an increased risk of acute liver injury during periods of Orlistat treatment compared to non-treatment periods (Douglas et al., 2013). Interestingly, preclinical studies have suggested a potentially beneficial hepatic effect in a specific context; in Gunn rats, a model for unconjugated hyperbilirubinemia, dietary Orlistat supplementation was found to effectively decrease plasma bilirubin levels (Hafkamp et al., 2005).

#### 5.2.2 Acute oxalate nephropathy

A rare but well-documented risk associated with Orlistat is the development of acute oxalate nephropathy. The underlying mechanism involves Orlistat-induced fat malabsorption. The resulting excess of unabsorbed free fatty acids in the intestinal lumen binds to dietary calcium, a process known as saponification. This reduces the amount of available calcium to bind with dietary oxalate, leading to increased absorption of free oxalate, subsequent hyperoxaluria, and the potential for calcium oxalate crystal deposition in the renal tubules, which can cause acute kidney injury (Ferraz et al., 2004; Singh et al., 2007). This risk is heightened in individuals with underlying chronic kidney disease or conditions predisposing to dehydration (Singh et al., 2007).

#### 5.2.3 Colonic effects and colorectal cancer risk

The increased delivery of fecal fat to the colon raised a theoretical concern about an altered colonic environment that could promote carcinogenesis. This hypothesis was supported by an early animal study in which rats treated with a chemical carcinogen and Orlistat showed an increase in colonic preneoplastic markers, specifically aberrant crypt foci (ACF) (Garcia et al., 2006). However, this finding has not been substantiated by human data. A clinical study in obese subjects found that 6 weeks of Orlistat treatment did not adversely affect colonic mucosal cell proliferation rates (Ahnen et al., 2007). More definitively, a large retrospective matched cohort study using UK primary care data, involving over 33,000 Orlistat initiators, found no association between Orlistat use and an increased risk of colorectal cancer (Hong et al., 2013). Further complexity is added by Orlistat’s systemic activity as an inhibitor of FASN, an enzyme upregulated in many cancers. FASN inhibition has been shown to impair angiogenesis and overcome tumor metabolic adaptations, suggesting a potential anti-neoplastic effect that might counteract any theoretical pro-carcinogenic luminal effects (Sounni et al., 2014; Bruning et al., 2018).

#### 5.2.4 Other systemic considerations

The metabolic changes induced by significant weight loss can have wide-ranging effects. For example, a large population-based cohort study found that weight loss following treatment with anti-obesity medications, including Orlistat, was associated with a modified risk of incident gout and recurrent gout flares (Wei et al., 2025).

### 5.3 Drug and nutrient interactions

The primary interactions of clinical significance involve the absorption of fat-soluble substances.

#### 5.3.1 Fat-soluble vitamins

By inhibiting fat digestion, Orlistat can reduce the absorption of fat-soluble vitamins (A, D, E, and K) and beta-carotene. Long-term clinical trials have consistently shown modest reductions in the plasma concentrations of these vitamins. Consequently, it is recommended that patients taking Orlistat also take a multivitamin supplement containing these vitamins, timed at least 2 hours apart from the Orlistat dose to ensure adequate absorption. The essentiality of lipid mobilization for fundamental biological processes, such as embryonic development in diverse species, underscores the importance of ensuring adequate nutrient status when pharmacologically targeting lipid metabolism (Itoe et al., 2024).

#### 5.3.2 Cyclosporine

A clinically significant drug interaction has been reported with the immunosuppressant cyclosporine. Orlistat can reduce the intestinal absorption of cyclosporine, leading to decreased plasma concentrations. A case report detailed the difficulty in achieving therapeutic cyclosporine levels in a renal transplant patient after initiating Orlistat, necessitating intravenous administration and careful dose titration (Evans et al., 2003). Co-administration requires close monitoring of cyclosporine blood levels to prevent potential allograft rejection.

### 5.4 Safety in special populations

Orlistat has been studied in adolescents aged 12–16 years. Its safety profile in this population is largely consistent with that observed in adults, with GI-related adverse events being the most common. Systematic reviews of weight management interventions in children and adolescents confirm this safety profile, though long-term data remain more limited than in adults (O’Connor et al., 2024). It is important to note that the generalizability of safety data for Orlistat may be influenced by the demographic characteristics of the populations studied in pivotal clinical trials (Alsaqaaby et al., 2024).

## 6 Emerging therapeutic applications: Orlistat in oncology

The repurposing of established drugs for oncology represents a promising and accelerated pathway for therapeutic development (Table 6). Orlistat, an FDA-approved inhibitor of pancreatic and gastric lipases used for obesity management (Yanovski and Yanovski, 2014), has attracted increasing interest as a potential anticancer agent (Figure 2). The rationale for this application is rooted in the profound metabolic reprogramming that characterizes many malignancies. Beyond the well-known Warburg effect, numerous cancer types exhibit a heightened dependence on *de novo* lipogenesis to supply fatty acids for rapid membrane biogenesis, energy storage, and the generation of signaling molecules (Tadros et al., 2017; Bu et al., 2024). This “lipogenic phenotype” is primarily driven by the overexpression and hyperactivity of FASN, a multi-enzyme complex that synthesizes palmitate from acetyl-CoA and malonyl-CoA. High FASN expression is frequently correlated with tumor aggressiveness and poor patient prognosis (Kridel et al., 2004). For instance, in acute myeloid leukemia (AML), the oncogenic driver FLT3/ITD has been shown to upregulate FASN via the SREBP transcription factors, directly linking a key cancer mutation to this metabolic vulnerability (Yin et al., 2025). Orlistat has also been shown to inhibit the thioesterase domain of FASN, thereby interfering with the final step of *de novo* fatty acid synthesis (Kridel et al., 2004). Collectively, these mechanistic observations provide a rationale for investigating Orlistat as a metabolic intervention in cancer; however, its potential anticancer activity remains largely supported by preclinical studies and should be distinguished from clinically established therapeutic efficacy.

**TABLE 6:** Orlistat in preclinical oncology studies.

Cancer type	Model	Mechanism	Main effect	References
Breast cancer	Cell lines	FASN inhibition, Her2 repression	Induce apoptosis and G1-S arrest	Kridel et al. (2004)
Prostate cancer	Cell lines and PC-3 xenograft tumor model	FASN inhibition	Halt tumor cell proliferation and induce tumor cell apoptosis	Kridel et al. (2004)
Pancreatic adenocarcinoma	Cell lines and orthotopic implantation models	Endoplasmic reticulum (ER) stress	Diminished stemness and resulted in apoptosis	Tadros et al. (2017)
B-cell chronic lymphocytic leukemia (B-CLL)	Cell lines	FASN inhibition	Sensitized leukemia cells to ABT-737	Samudio et al. (2010)
Hepatocellular carcinoma (HCC)	Mouse model	FASN inhibition, PD-L1 blockade	Immune-mediated tumor killing	Huang et al. (2024)

**FIGURE 2:**
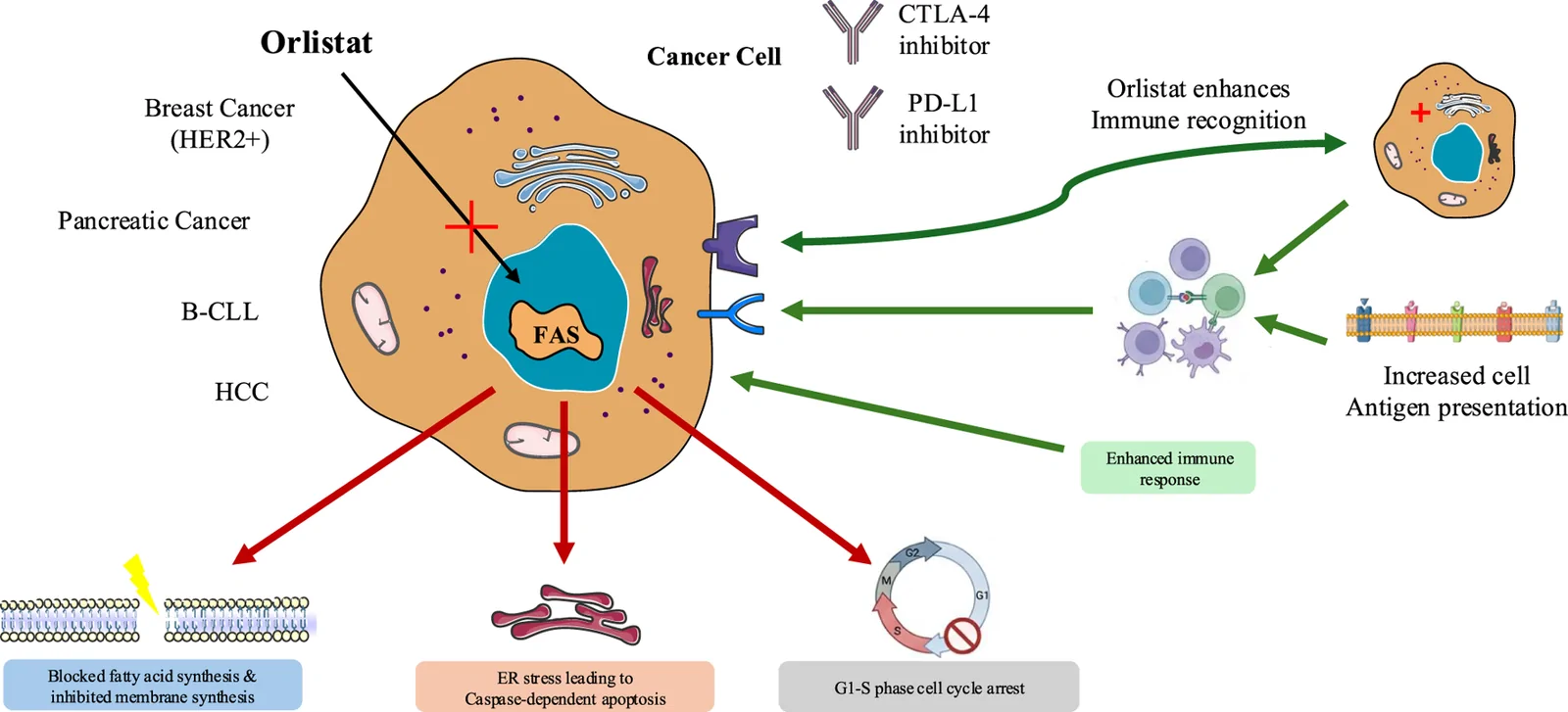
Antitumor mechanisms of Orlistat via FASN inhibition. In cancer cells, Orlistat blocks fatty acid synthesis, induces ER stress, apoptosis, and cell cycle arrest. Combination with immunotherapy (CTLA-4/PD-L1 blockade) further enhances antitumor effects.

Preclinical studies have provided consistent evidence supporting the potential anti-neoplastic activity of Orlistat. Initial investigations demonstrated its efficacy in breast and prostate cancer cell lines, which frequently overexpress FASN. This has been expanded to include pancreatic adenocarcinoma, a malignancy where altered lipid metabolism is a significant prognostic factor (Tadros et al., 2017). The tumor microenvironment in pancreatic cancer is rich in lipids, and the pathological accumulation of unsaturated fatty acids from peripancreatic fat necrosis can exacerbate disease severity, highlighting the critical role of lipid metabolism in this disease (Noel et al., 2016). In hematological malignancies, Orlistat has been shown to target lipoprotein lipase (LPL), another enzyme overexpressed in B-cell chronic lymphocytic leukemia (B-CLL), resulting in the induction of apoptosis. This finding underscores that targeting lipid metabolic pathways, whether synthesis via FASN or uptake via LPL, is a valid therapeutic strategy. Indeed, the broader concept of exploiting lipid metabolism as a dependency has been validated by studies showing that inhibition of fatty acid oxidation can also sensitize leukemia cells to apoptosis (Samudio et al., 2010).

The molecular mechanisms underlying Orlistat’s anti-cancer effects are multifaceted. The primary consequence of FASN inhibition is “lipid starvation,” which triggers several downstream cytotoxic pathways. A key mechanism is the induction of profound endoplasmic reticulum (ER) stress. By depleting the supply of newly synthesized fatty acids required for phospholipid production and membrane integrity, FASN inhibition disrupts ER homeostasis, leading to the activation of the unfolded protein response and subsequent apoptosis. Furthermore, Orlistat treatment has been shown to induce G1-S phase cell cycle arrest and promote apoptotic cell death through caspase activation in breast cancer cells. In HER2-positive breast cancer, FASN inhibition by Orlistat was also linked to the PEA3-mediated transcriptional repression of the *Her2/neu* (*erbB-2*) oncogene, suggesting a direct impact on oncogenic signaling pathways.

An emerging area of investigation is the potential use of Orlistat in combination with established anticancer therapies to overcome resistance and enhance the efficacy of existing treatments (Table 7). The metabolic flexibility of tumors allows them to adapt to therapies; for instance, resistance to antiangiogenic agents like sunitinib can involve a metabolic shift toward increased lipid metabolism, a vulnerability that can be exploited by co-administering a lipid synthesis inhibitor (Sounni et al., 2014). Similarly, Orlistat has been shown to resensitize tamoxifen-resistant breast cancer cells by inhibiting the formation of lipid droplets, which play a crucial role in drug resistance and cancer progression (Park et al., 2025). More recently, a strong rationale has emerged for combining Orlistat with modern immunotherapies. Preclinical models demonstrate that Orlistat can potentiate the effects of CTLA-4 blockade by suppressing the expression of the immune checkpoint protein PD-L1 through an AKT-FOXO3a-FOXM1 signaling axis (Tang et al., 2025). In hepatocellular carcinoma (HCC), pharmacological inhibition of FASN was found to synergize with PD-L1 checkpoint blockade by preventing the degradation of MHC-I molecules on cancer cells, thereby enhancing antigen presentation and cytotoxic T-cell recognition (Huang et al., 2024). These findings suggest that by modulating the metabolic state of tumor cells, Orlistat can render them more visible and susceptible to the immune system. Future clinical translation may be aided by novel drug delivery strategies, such as Orlistat-loaded metal-organic frameworks designed to suppress fatty acid metabolism and induce cuproptosis, a distinct form of cell death, to synergistically treat cancer lymph node metastasis (Li Z. Z. et al., 2025). As obesity is a major risk factor for many cancers, repurposing an anti-obesity drug like Orlistat offers the tantalizing prospect of simultaneously targeting a systemic risk factor and a core metabolic vulnerability of the tumor itself. In contrast to its established metabolic indications, the anticancer evidence remains largely preclinical and requires clinical validation.

**TABLE 7:** Combination strategies involving Orlistat in cancer models.

Cancer	Combination	Mechanism	Evidence	References
HCC	Orlistat + anti-PD-L1	Enhanced antigen presentation	Mouse	Huang et al. (2024)
HCC	Orlistat + CTLA-4 blockade	PD-L1 suppression	Mouse	Tang et al. (2025)
Breast cancer	Orlistat + tamoxifen	Lipid droplet inhibition	Cell model	Park et al. (2025)
CLL	Orlistat + ABT-737	Metabolic vulnerability	Cell model	Samudio et al. (2010)

Despite these encouraging preclinical findings, several limitations should be considered when evaluating Orlistat for oncology. Most evidence remains derived from cancer cell lines and animal models, with limited clinical validation. In addition, the limited systemic exposure of Orlistat may restrict its delivery to tumor tissues and intracellular targets such as FASN, and the concentrations used in some preclinical models may not be readily achievable in patients. Potential off-target effects and the lack of complete tumor selectivity of FASN inhibition may further limit its therapeutic window (Cui et al., 2026). Importantly, the safety profile established in obesity may not be directly applicable to patients with cancer, particularly those with malnutrition or cachexia. Therefore, targeted delivery, optimized formulations, or more selective FASN-directed strategies may be required to overcome these translational barriers.

## 7 Conclusion and future perspectives

Orlistat has maintained a durable position in the therapeutic arsenal for obesity management for over 2 decades. This review reaffirms its status as a moderately effective agent for long-term weight reduction and maintenance, particularly when integrated with comprehensive lifestyle modifications encompassing diet and physical activity (Sjostrom et al., 1998; Davidson et al., 1999). Its mechanism, the inhibition of gastric and pancreatic lipases, directly reduces dietary fat absorption, which not only facilitates weight loss but also confers significant benefits on metabolic health. Extensive clinical evidence has demonstrated that Orlistat therapy is associated with improvements in glycemic control, reductions in LDL cholesterol and blood pressure, and a decreased incidence of type 2 diabetes in at-risk populations (Miles et al., 2002; Kelley et al., 2004; Sahebkar et al., 2017). In an era increasingly dominated by highly effective incretin-based therapies, Orlistat’s distinct, non-systemic mechanism of action and its availability as an over-the-counter medication secure its niche as a viable option for certain patient populations (Shi et al., 2024; McGowan et al., 2025). However, the primary clinical challenge remains the management of its predictable, mechanism-based gastrointestinal adverse effects, such as steatorrhea, flatulence, and fecal urgency, which frequently lead to poor patient adherence and treatment discontinuation, thereby limiting its real-world effectiveness (Johansson et al., 2009).

Looking forward, the most compelling avenue for future research lies beyond Orlistat’s role in obesity and extends into the realm of oncology. A substantial body of preclinical evidence has repurposed Orlistat as a potent, irreversible inhibitor of FASN, a key enzyme in *de novo* lipogenesis (Kridel et al., 2004; Pemble et al., 2007). FASN is minimally expressed in most normal adult tissues but is significantly upregulated in numerous human cancers, including breast, prostate, and colorectal cancers, where its expression often correlates with tumor aggressiveness and poor prognosis (Menendez et al., 2005; Wei et al., 2023). The inhibition of FASN by Orlistat has been shown to block cell cycle progression, induce apoptosis in cancer cells, and even repress the expression of key oncogenes like Her2/neu (Menendez et al., 2005). This connection between lipid metabolism and cancer is being actively explored, with recent studies linking obesity-related genes to pancreatic cancer progression via fatty acid elongation pathways (Chu et al., 2025) and demonstrating the role of FASN-mediated lipogenesis in pathologies like periodontitis (Xiao et al., 2025) and *Fusobacterium* nucleatum-infected colorectal cancer (Zhou et al., 2025). This suggests that targeting FASN could have broad therapeutic implications.

The future development and clinical validation of Orlistat and its analogues will likely proceed along several parallel tracks. Firstly, there is an urgent need for well-designed clinical trials to translate the promising preclinical anti-cancer data into human therapies. These studies must evaluate the efficacy of Orlistat, either as a monotherapy or in combination with standard chemotherapeutics or immunotherapies, in FASN-overexpressing tumors. Secondly, overcoming the challenge of gastrointestinal side effects remains a priority. This could be achieved through the development of novel formulations, such as lipid nanoemulsions designed for prolonged or targeted release (Baumann et al., 2024), or through co-therapies that mitigate lipase inhibition-related symptoms. The ongoing search for novel pancreatic lipase inhibitors from natural and synthetic sources, which often use Orlistat as a benchmark, underscores the demand for alternatives with improved tolerability (Cao et al., 2025; Li Y. et al., 2025). Thirdly, continued long-term pharmacovigilance is essential to monitor the safety profile of Orlistat, particularly concerning rare but serious adverse events like acute liver injury and oxalate nephropathy (Singh et al., 2007; Douglas et al., 2013). Finally, further research is warranted to assess its utility in specific populations, such as youth living with obesity, where therapeutic options remain limited. In conclusion, while Orlistat’s role in obesity management is well-established, its future potential as a repurposed FASN inhibitor in oncology represents a transformative area for research that may broaden its potential therapeutic applications if its pharmacokinetic and translational limitations can be overcome.

More broadly, the pharmacological trajectory of Orlistat illustrates how established drugs can provide mechanistic links between metabolism and disease and serve as starting points for therapeutic repurposing. Continued integration of pharmacology, metabolic biology, and oncology may help identify new applications for established compounds while also clarifying the limitations that must be addressed before preclinical observations can be translated into clinical benefit.

## References

[B1] Ahnen D. J. Guerciolini R. Hauptman J. Blotner S. Woods C. J. Wargovich M. J. (2007). Effect of orlistat on fecal fat, fecal biliary acids, and colonic cell proliferation in obese subjects. Clin. Gastroenterol. Hepatol. 5 (11), 1291–1299. 10.1016/j.cgh.2007.07.009 17920338

[B2] Aldekhail N. M. Logue J. McLoone P. Morrison D. S. (2015). Effect of orlistat on glycaemic control in overweight and obese patients with type 2 diabetes mellitus: a systematic review and meta-analysis of randomized controlled trials. Obes. Rev. 16 (12), 1071–1080. 10.1111/obr.12318 26345590

[B3] Alsaqaaby M. S. Cooney S. le Roux C. W. Pournaras D. J. (2024). Sex, race, and BMI in clinical trials of medications for obesity over the past three decades: a systematic review. Lancet Diabetes Endocrinol. 12 (6), 414–421. 10.1016/S2213-8587(24)00098-6 38723646

[B4] Astrup A. Rossner S. Van Gaal L. Rissanen A. Niskanen L. Al Hakim M. (2009). Effects of liraglutide in the treatment of obesity: a randomised, double-blind, placebo-controlled study. Lancet 374 (9701), 1606–1616. 10.1016/S0140-6736(09)61375-1 19853906

[B5] Baumann N. Baumgarten J. Kunick C. Bunjes H. (2024). Prolonged release from lipid nanoemulsions by modification of drug lipophilicity. J. Control Release 374, 478–488. 10.1016/j.jconrel.2024.08.021 39151830

[B6] Bello M. Basilio-Antonio L. Fragoso-Vazquez J. Avalos-Soriano A. Correa-Basurto J. (2017). Molecular recognition between pancreatic lipase and natural and synthetic inhibitors. Int. J. Biol. Macromol. 98, 855–868. 10.1016/j.ijbiomac.2017.01.150 28212930

[B7] Bergholm R. Tiikkainen M. Vehkavaara S. Tamminen M. Teramo K. Rissanen A. (2003). Lowering of LDL cholesterol rather than moderate weight loss improves endothelium-dependent vasodilatation in obese women with previous gestational diabetes. Diabetes Care 26 (6), 1667–1672. 10.2337/diacare.26.6.1667 12766091

[B8] Borovicka J. Schwizer W. Guttmann G. Hartmann D. Kosinski M. Wastiel C. (2000). Role of lipase in the regulation of postprandial gastric acid secretion and emptying of fat in humans: a study with orlistat, a highly specific lipase inhibitor. Gut 46 (6), 774–781. 10.1136/gut.46.6.774 10807887 PMC1756442

[B9] Bruning U. Morales-Rodriguez F. Kalucka J. Goveia J. Taverna F. Queiroz K. C. S. (2018). Impairment of angiogenesis by fatty acid synthase inhibition involves mTOR malonylation. Cell Metab. 28 (6), 866–880 e815. 10.1016/j.cmet.2018.07.019 30146486 PMC8057116

[B10] Bu L. Zhang Z. Chen J. Fan Y. Guo J. Su Y. (2024). High-fat diet promotes liver tumorigenesis via palmitoylation and activation of AKT. Gut 73 (7), 1156–1168. 10.1136/gutjnl-2023-330826 38191266

[B11] Cao R. Li J. Sun Y. Li Y. Zhang X. L. Ding H. (2025). Synthesis, characterization, and *in vitro* hypolipidemic mechanisms of sugar-SA triazole conjugates as potent pancreatic lipase inhibitors. Food Chem. 488, 144876. 10.1016/j.foodchem.2025.144876 40413947

[B12] Chanoine J. P. Hampl S. Jensen C. Boldrin M. Hauptman J. (2005). Effect of orlistat on weight and body composition in obese adolescents: a randomized controlled trial. JAMA 293 (23), 2873–2883. 10.1001/jama.293.23.2873 15956632

[B13] Chu X. Zhao J. Shen Y. Feng Q. Zhou C. Ma L. (2025). HACD2 promotes pancreatic cancer progression by enhancing PKM2 dissociation from PRKN in a dehydratase-independent manner. Adv. Sci. (Weinh) 12 (10), e2407942. 10.1002/advs.202407942 39836601 PMC11904967

[B14] Cui Q. Josephraj S. Gu B. Liu J. Y. Zhang J. T. (2026). Targeting fatty acid synthase for cancer drug discovery: retrospective analyses and outlook. Pharmacol. Rev. 78 (1), 100105. 10.1016/j.pharmr.2025.100105 41519050 PMC13169002

[B15] Czernichow S. Lee C. M. Barzi F. Greenfield J. R. Baur L. A. Chalmers J. (2010). Efficacy of weight loss drugs on obesity and cardiovascular risk factors in obese adolescents: a meta-analysis of randomized controlled trials. Obes. Rev. 11 (2), 150–158. 10.1111/j.1467-789X.2009.00620.x 19573052

[B16] Czernichow S. Knol M. J. Fezeu L. Grobbee D. E. (2012). Impact of orlistat initiation on cardiovascular treatment use: a 6-year population-based cohort study. Eur. J. Prev. Cardiol. 19 (3), 484–489. 10.1177/1741826711406058 21464100

[B17] Damci T. Yalin S. Balci H. Osar Z. Korugan U. Ozyazar M. (2004). Orlistat augments postprandial increases in glucagon-like peptide 1 in obese type 2 diabetic patients. Diabetes Care 27 (5), 1077–1080. 10.2337/diacare.27.5.1077 15111524

[B18] Davidson M. H. Hauptman J. DiGirolamo M. Foreyt J. P. Halsted C. H. Heber D. (1999). Weight control and risk factor reduction in obese subjects treated for 2 years with orlistat: a randomized controlled trial. JAMA 281 (3), 235–242. 10.1001/jama.281.3.235 9918478

[B19] Dombrowski S. U. Knittle K. Avenell A. Araujo-Soares V. Sniehotta F. F. (2014). Long term maintenance of weight loss with non-surgical interventions in obese adults: systematic review and meta-analyses of randomised controlled trials. BMJ 348, g2646. 10.1136/bmj.g2646 25134100 PMC4020585

[B20] Douglas I. J. Langham J. Bhaskaran K. Brauer R. Smeeth L. (2013). Orlistat and the risk of acute liver injury: self controlled case series study in UK clinical practice research datalink. BMJ 346, f1936. 10.1136/bmj.f1936 23585064 PMC3624963

[B21] Evans S. Michael R. Wells H. Maclean D. Gordon I. Taylor J. (2003). Drug interaction in a renal transplant patient: cyclosporin-neoral and orlistat. Am. J. Kidney Dis. 41 (2), 493–496. 10.1053/ajkd.2003.50061 12552515

[B22] Fabricatore A. N. Wadden T. A. Moore R. H. Butryn M. L. Gravallese E. A. Erondu N. E. (2009). Attrition from randomized controlled trials of pharmacological weight loss agents: a systematic review and analysis. Obes. Rev. 10 (3), 333–341. 10.1111/j.1467-789X.2009.00567.x 19389060 PMC2682632

[B23] Fako V. E. Zhang J. T. Liu J. Y. (2014). Mechanism of orlistat hydrolysis by the thioesterase of human fatty acid synthase. ACS Catal. 4 (10), 3444–3453. 10.1021/cs500956m 25309810 PMC4188697

[B24] Feinle C. Rades T. Otto B. Fried M. (2001). Fat digestion modulates gastrointestinal sensations induced by gastric distention and duodenal lipid in humans. Gastroenterology 120 (5), 1100–1107. 10.1053/gast.2001.23232 11266374

[B25] Ferraz R. R. Tiselius H. G. Heilberg I. P. Heiberg I. P. (2004). Fat malabsorption induced by gastrointestinal lipase inhibitor leads to an increase in urinary oxalate excretion. Kidney Int. 66 (2), 676–682. 10.1111/j.1523-1755.2004.00790.x 15253722

[B26] Garcia S. B. Barros L. T. Turatti A. Martinello F. Modiano P. Ribeiro-Silva A. (2006). The anti-obesity agent orlistat is associated to increase in colonic preneoplastic markers in rats treated with a chemical carcinogen. Cancer Lett. 240 (2), 221–224. 10.1016/j.canlet.2005.09.011 16377080

[B27] Gillies C. L. Abrams K. R. Lambert P. C. Cooper N. J. Sutton A. J. Hsu R. T. (2007). Pharmacological and lifestyle interventions to prevent or delay type 2 diabetes in people with impaired glucose tolerance: systematic review and meta-analysis. BMJ 334 (7588), 299. 10.1136/bmj.39063.689375.55 17237299 PMC1796695

[B28] Goldberg A. Graca S. Liu J. Rao V. Witchel S. F. Pena A. (2024). Anti-obesity pharmacological agents for polycystic ovary syndrome: a systematic review and meta-analysis to inform the 2023 international evidence-based guideline. Obes. Rev. 25 (5), e13704. 10.1111/obr.13704 38355887

[B29] Grilo C. M. Masheb R. M. Salant S. L. (2005). Cognitive behavioral therapy guided self-help and orlistat for the treatment of binge eating disorder: a randomized, double-blind, placebo-controlled trial. Biol. Psychiatry 57 (10), 1193–1201. 10.1016/j.biopsych.2005.03.001 15866560

[B30] Grunvald E. Shah R. Hernaez R. Chandar A. K. Pickett-Blakely O. Teigen L. M. (2022). AGA clinical practice guideline on pharmacological interventions for adults with obesity. Gastroenterology 163 (5), 1198–1225. 10.1053/j.gastro.2022.08.045 36273831

[B31] Hafkamp A. M. Havinga R. Sinaasappel M. Verkade H. J. (2005). Effective oral treatment of unconjugated hyperbilirubinemia in Gunn rats. Hepatology 41 (3), 526–534. 10.1002/hep.20589 15726662

[B32] Harrison S. Ramrakhiani S. Brunt E. M. Anbari M. A. Cortese C. Bacon B. R. (2003). Orlistat in the treatment of NASH: a case series. Am. J. Gastroenterology 98 (4), 926–930. 10.1111/j.1572-0241.2003.07375.x 12738478

[B33] Harrison S. A. Fecht W. Brunt E. M. Neuschwander-Tetri B. A. (2009). Orlistat for overweight subjects with nonalcoholic steatohepatitis: a randomized, prospective trial. Hepatology 49 (1), 80–86. 10.1002/hep.22575 19053049

[B34] Hollander P. A. Elbein S. C. Hirsch I. B. Kelley D. McGill J. Taylor T. (1998). Role of orlistat in the treatment of obese patients with type 2 diabetes. A 1-year randomized double-blind study. Diabetes Care 21 (8), 1288–1294. 10.2337/diacare.21.8.1288 9702435

[B35] Hong J. L. Meier C. R. Sandler R. S. Jick S. S. Sturmer T. (2013). Risk of colorectal cancer after initiation of orlistat: matched cohort study. BMJ 347, f5039. 10.1136/bmj.f5039 23982291 PMC3754767

[B36] Huang J. Tsang W. Y. Fang X. N. Zhang Y. Luo J. Gong L. Q. (2024). FASN inhibition decreases MHC-I degradation and synergizes with PD-L1 checkpoint blockade in hepatocellular carcinoma. Cancer Res. 84 (6), 855–871. 10.1158/0008-5472.CAN-23-0966 38486485

[B37] Itoe M. A. Shaw W. R. Stryapunina I. Vidoudez C. Peng D. Du E. W. (2024). Maternal lipid mobilization is essential for embryonic development in the malaria vector Anopheles gambiae. PLoS Biol. 22 (12), e3002960. 10.1371/journal.pbio.3002960 39689130 PMC11703037

[B38] Johansson K. Neovius K. DeSantis S. M. Rossner S. Neovius M. (2009). Discontinuation due to adverse events in randomized trials of orlistat, sibutramine and rimonabant: a meta-analysis. Obes. Rev. 10 (5), 564–575. 10.1111/j.1467-789X.2009.00581.x 19460116

[B39] Kelley D. E. Bray G. A. Pi-Sunyer F. X. Klein S. Hill J. Miles J. (2002). Clinical efficacy of orlistat therapy in overweight and obese patients with insulin-treated type 2 diabetes: a 1-year randomized controlled trial. Diabetes Care 25 (6), 1033–1041. 10.2337/diacare.25.6.1033 12032111

[B40] Kelley D. E. Kuller L. H. McKolanis T. M. Harper P. Mancino J. Kalhan S. (2004). Effects of moderate weight loss and orlistat on insulin resistance, regional adiposity, and fatty acids in type 2 diabetes. Diabetes Care 27 (1), 33–40. 10.2337/diacare.27.1.33 14693963

[B41] Khera R. Murad M. H. Chandar A. K. Dulai P. S. Wang Z. Prokop L. J. (2016). Association of pharmacological treatments for obesity with weight loss and adverse events: a systematic review and meta-analysis. JAMA 315 (22), 2424–2434. 10.1001/jama.2016.7602 27299618 PMC5617638

[B42] Khera R. Pandey A. Chandar A. K. Murad M. H. Prokop L. J. Neeland I. J. (2018). Effects of weight-loss medications on cardiometabolic risk profiles: a systematic review and network meta-analysis. Gastroenterology 154 (5), 1309–1319 e1307. 10.1053/j.gastro.2017.12.024 29305933 PMC5880739

[B43] Kimura W. Meyer F. Hess D. Kirchner T. Fischbach W. Mossner J. (1992). Comparison of different treatment modalities in experimental pancreatitis in rats. Gastroenterology 103 (6), 1916–1924. 10.1016/0016-5085(92)91452-a 1451985

[B44] Kitadokoro K. Tanaka M. Hikima T. Okuno Y. Yamamoto M. Kamitani S. (2020). Crystal structure of pathogenic *Staphylococcus aureus* lipase complex with the anti-obesity drug orlistat. Sci. Rep. 10 (1), 5469. 10.1038/s41598-020-62427-8 32214208 PMC7096528

[B45] Kridel S. J. Axelrod F. Rozenkrantz N. Smith J. W. (2004). Orlistat is a novel inhibitor of fatty acid synthase with antitumor activity. Cancer Res. 64 (6), 2070–2075. 10.1158/0008-5472.can-03-3645 15026345

[B46] Lamotte M. Annemans L. Lefever A. Nechelput M. Masure J. (2002). A health economic model to assess the long-term effects and cost-effectiveness of orlistat in obese type 2 diabetic patients. Diabetes Care 25 (2), 303–308. 10.2337/diacare.25.2.303 11815500

[B47] Leblanc E. S. O'Connor E. Whitlock E. P. Patnode C. D. Kapka T. (2011). Effectiveness of primary care-relevant treatments for obesity in adults: a systematic evidence review for the U.S. preventive services task force. Ann. Intern Med. 155 (7), 434–447. 10.7326/0003-4819-155-7-201110040-00006 21969342

[B48] Li Z. Maglione M. Tu W. Mojica W. Arterburn D. Shugarman L. R. (2005). Meta-analysis: pharmacologic treatment of obesity. Ann. Intern Med. 142 (7), 532–546. 10.7326/0003-4819-142-7-200504050-00012 15809465

[B49] Li Y. Zhang Y. Zhang L. Dang J. (2025). Bioactivity-guided isolation of pancreatic lipase inhibitors from Lycium ruthenicum using a novel HPLC-FLD/pancreatic lipase online recognition system. Food Chem. 497, 147031. 10.1016/j.foodchem.2025.147031 41223526

[B50] Li Z. Z. Liu Y. Zhou K. Cao L. M. Wang G. R. Wu J. (2025). ORL@Cu-MOF boost cuproptosis and suppress fatty acid metabolism for cancer lymph node metastasis synergistic therapy. Adv. Sci. (Weinh) 12 (35), e02154. 10.1002/advs.202502154 40548889 PMC12463119

[B51] Lindgarde F. (2000). The effect of orlistat on body weight and coronary heart disease risk profile in obese patients: the Swedish multimorbidity study. J. Intern Med. 248 (3), 245–254. 10.1046/j.1365-2796.2000.00720.x 10971792

[B52] Little J. L. Wheeler F. B. Fels D. R. Koumenis C. Kridel S. J. (2007). Inhibition of fatty acid synthase induces endoplasmic reticulum stress in tumor cells. Cancer Res. 67 (3), 1262–1269. 10.1158/0008-5472.CAN-06-1794 17283163

[B53] MacLaughlin H. L. Cook S. A. Kariyawasam D. Roseke M. van Niekerk M. Macdougall I. C. (2010). Nonrandomized trial of weight loss with orlistat, nutrition education, diet, and exercise in obese patients with CKD: 2-year follow-up. Am. J. Kidney Dis. 55 (1), 69–76. 10.1053/j.ajkd.2009.09.011 19926371

[B54] Madsen E. L. Bruun J. M. Skogstrand K. Hougaard D. M. Christiansen T. Richelsen B. (2009). Long-term weight loss decreases the nontraditional cardiovascular risk factors interleukin-18 and matrix metalloproteinase-9 in obese subjects. Metabolism 58 (7), 946–953. 10.1016/j.metabol.2009.02.031 19409578

[B55] McDowell K. Petrie M. C. Raihan N. A. Logue J. (2018). Effects of intentional weight loss in patients with obesity and heart failure: a systematic review. Obes. Rev. 19 (9), 1189–1204. 10.1111/obr.12707 30051959

[B56] McGowan B. Ciudin A. Baker J. L. Busetto L. Dicker D. Fruhbeck G. (2025). A systematic review and meta-analysis of the efficacy and safety of pharmacological treatments for obesity in adults. Nat. Med. 31 (10), 3317–3329. 10.1038/s41591-025-03978-z 41039116 PMC12532627

[B57] Menendez J. A. Vellon L. Lupu R. (2005). Antitumoral actions of the anti-obesity drug orlistat (XenicalTM) in breast cancer cells: blockade of cell cycle progression, promotion of apoptotic cell death and PEA3-mediated transcriptional repression of Her2/neu (erbB-2) oncogene. Ann. Oncol. 16 (8), 1253–1267. 10.1093/annonc/mdi239 15870086

[B58] Miles J. M. Leiter L. Hollander P. Wadden T. Anderson J. W. Doyle M. (2002). Effect of orlistat in overweight and obese patients with type 2 diabetes treated with metformin. Diabetes Care 25 (7), 1123–1128. 10.2337/diacare.25.7.1123 12087008

[B59] Mulzer M. Tiegs B. J. Wang Y. Coates G. W. O'Doherty G. A. (2014). Total synthesis of tetrahydrolipstatin and stereoisomers via a highly regio- and diastereoselective carbonylation of epoxyhomoallylic alcohols. J. Am. Chem. Soc. 136 (30), 10814–10820. 10.1021/ja505639u 25004122 PMC4120994

[B60] Noel P. Patel K. Durgampudi C. Trivedi R. N. de Oliveira C. Crowell M. D. (2016). Peripancreatic fat necrosis worsens acute pancreatitis independent of pancreatic necrosis via unsaturated fatty acids increased in human pancreatic necrosis collections. Gut 65 (1), 100–111. 10.1136/gutjnl-2014-308043 25500204 PMC4869971

[B61] O'ConnorE. A. EvansC. V. BurdaB. U. WalshE. S. EderM. LozanoP. (2017). Screening for obesity and intervention for weight management in children and adolescents: evidence report and systematic review for the US preventive services task force. JAMA 317 (23), 2427–2444. 10.1001/jama.2017.0332 28632873

[B62] O'ConnorE. A. EvansC. V. HenningerM. RedmondN. SengerC. A. (2024). Interventions for weight management in children and adolescents: updated evidence report and systematic review for the US preventive services task force. JAMA 332 (3), 233–248. 10.1001/jama.2024.6739 38888913

[B63] Padwal R. Majumdar S. R. Johnson J. A. Varney J. McAlister F. A. (2005). A systematic review of drug therapy to delay or prevent type 2 diabetes. Diabetes Care 28 (3), 736–744. 10.2337/diacare.28.3.736 15735219

[B64] Pallasch C. P. Schwamb J. Konigs S. Schulz A. Debey S. Kofler D. (2008). Targeting lipid metabolism by the lipoprotein lipase inhibitor orlistat results in apoptosis of B-cell chronic lymphocytic leukemia cells. Leukemia 22 (3), 585–592. 10.1038/sj.leu.2405058 18079738

[B65] Park J. Y. Lee J. Kim Y. Y. Lee S. Kim Y. (2025). Synergistic combination of tamoxifen-tetraphenylethylene Co-Assembled micelles and Orlistat-Induced lipid droplet inhibition to overcome tamoxifen resistance. Adv. Healthc. Mater 14 (32), e02060. 10.1002/adhm.202502060 40801177 PMC12716221

[B66] Peck K. H. Dulay M. S. Hameed S. Rosano G. Tan T. Dar O. (2024). Intentional weight loss in overweight and obese patients with heart failure: a systematic review. Eur. J. Heart Fail 26 (9), 1907–1930. 10.1002/ejhf.3270 38752254

[B67] Pemble C. W. t. Johnson L. C. Kridel S. J. Lowther W. T. (2007). Crystal structure of the thioesterase domain of human fatty acid synthase inhibited by orlistat. Nat. Struct. Mol. Biol. 14 (8), 704–709. 10.1038/nsmb1265 17618296

[B68] Peng L. Wang J. Li F. (2011). Weight reduction for non-alcoholic fatty liver disease. Cochrane Database Syst. Rev. 6, CD003619. 10.1002/14651858.CD003619.pub3 21678341

[B69] Reitsma J. B. Castro Cabezas M. de Bruin T. W. Erkelens D. W. (1994). Relationship between improved postprandial lipemia and low-density lipoprotein metabolism during treatment with tetrahydrolipstatin, a pancreatic lipase inhibitor. Metabolism 43 (3), 293–298. 10.1016/0026-0495(94)90095-7 8139476

[B70] Richelsen B. Tonstad S. Rossner S. Toubro S. Niskanen L. Madsbad S. (2007). Effect of orlistat on weight regain and cardiovascular risk factors following a very-low-energy diet in abdominally obese patients: a 3-year randomized, placebo-controlled study. Diabetes Care 30 (1), 27–32. 10.2337/dc06-0210 17192328

[B71] Rucker D. Padwal R. Li S. K. Curioni C. Lau D. C. (2007). Long term pharmacotherapy for obesity and overweight: updated meta-analysis. BMJ 335 (7631), 1194–1199. 10.1136/bmj.39385.413113.25 18006966 PMC2128668

[B72] Sahebkar A. Simental-Mendia L. E. Reiner Z. Kovanen P. T. Simental-Mendia M. Bianconi V. (2017). Effect of orlistat on plasma lipids and body weight: a systematic review and meta-analysis of 33 randomized controlled trials. Pharmacol. Res. 122, 53–65. 10.1016/j.phrs.2017.05.022 28559211

[B73] Samudio I. Harmancey R. Fiegl M. Kantarjian H. Konopleva M. Korchin B. (2010). Pharmacologic inhibition of fatty acid oxidation sensitizes human leukemia cells to apoptosis induction. J. Clin. Invest. 120 (1), 142–156. 10.1172/JCI38942 20038799 PMC2799198

[B74] Schroll J. B. Penninga E. I. Gotzsche P. C. (2016). Assessment of adverse events in protocols, clinical study reports, and published papers of trials of orlistat: a document analysis. PLoS Med. 13 (8), e1002101. 10.1371/journal.pmed.1002101 27529343 PMC4987052

[B75] Shi Q. Wang Y. Hao Q. Vandvik P. O. Guyatt G. Li J. (2024). Pharmacotherapy for adults with overweight and obesity: a systematic review and network meta-analysis of randomised controlled trials. Lancet 403 (10434), e21–e31. 10.1016/S0140-6736(24)00351-9 38582569

[B76] Singh A. Sarkar S. R. Gaber L. W. Perazella M. A. (2007). Acute oxalate nephropathy associated with orlistat, a gastrointestinal lipase inhibitor. Am. J. Kidney Dis. 49 (1), 153–157. 10.1053/j.ajkd.2006.10.004 17185156

[B77] Sjostrom L. Rissanen A. Andersen T. Boldrin M. Golay A. Koppeschaar H. P. (1998). Randomised placebo-controlled trial of orlistat for weight loss and prevention of weight regain in obese patients. European multicentre orlistat study group. Lancet 352 (9123), 167–172. 10.1016/s0140-6736(97)11509-4 9683204

[B78] Slebe F. Rojo F. Vinaixa M. Garcia-Rocha M. Testoni G. Guiu M. (2016). FoxA and LIPG endothelial lipase control the uptake of extracellular lipids for breast cancer growth. Nat. Commun. 7, 11199. 10.1038/ncomms11199 27045898 PMC4822041

[B79] Sounni N. E. Cimino J. Blacher S. Primac I. Truong A. Mazzucchelli G. (2014). Blocking lipid synthesis overcomes tumor regrowth and metastasis after antiangiogenic therapy withdrawal. Cell Metab. 20 (2), 280–294. 10.1016/j.cmet.2014.05.022 25017943

[B80] Tadros S. Shukla S. K. King R. J. Gunda V. Vernucci E. Abrego J. (2017). *De novo* Lipid Synthesis Facilitates Gemcitabine Resistance through Endoplasmic Reticulum Stress in Pancreatic Cancer. Cancer Res. 77 (20), 5503–5517. 10.1158/0008-5472.CAN-16-3062 28811332 PMC5645242

[B81] Tang Q. Li J. Zhang L. Zeng S. Bao Q. Hu W. (2025). Orlistat facilitates immunotherapy via AKT-FOXO3a-FOXM1-mediated PD-L1 suppression. J. Immunother. Cancer 13 (3), e008923. 10.1136/jitc-2024-008923 40139835 PMC11951015

[B82] Torgerson J. S. Hauptman J. Boldrin M. N. Sjostrom L. (2004). XENical in the prevention of diabetes in obese subjects (XENDOS) study: a randomized study of orlistat as an adjunct to lifestyle changes for the prevention of type 2 diabetes in obese patients. Diabetes Care 27 (1), 155–161. 10.2337/diacare.27.1.155 14693982

[B83] Wang F. F. Wu Y. Zhu Y. H. Ding T. Batterham R. L. Qu F. (2018). Pharmacologic therapy to induce weight loss in women who have obesity/overweight with polycystic ovary syndrome: a systematic review and network meta-analysis. Obes. Rev. 19 (10), 1424–1445. 10.1111/obr.12720 30066361

[B84] Wei W. Qin B. Wen W. Zhang B. Luo H. Wang Y. (2023). FBXW7beta loss-of-function enhances FASN-mediated lipogenesis and promotes colorectal cancer growth. Signal Transduct. Target Ther. 8 (1), 187. 10.1038/s41392-023-01405-8 37202390 PMC10195794

[B85] Wei J. Wang Y. Dalbeth N. Xie J. Wu J. Zeng C. (2025). Weight loss after receiving anti-obesity medications and gout among individuals with overweight and Obese: a population-based cohort study. Arthritis Rheumatol. 77 (3), 335–345. 10.1002/art.42996 39300602

[B86] Xiao Y. Ma J. Li Z. Gou H. Chen X. Zhou Y. (2025). NAMPT impairs vascular permeability in periodontitis by influencing FASN-mediated lipogenesis. Int. J. Biol. Sci. 21 (6), 2707–2724. 10.7150/ijbs.104485 40303304 PMC12035901

[B87] Yang P. Y. Liu K. Ngai M. H. Lear M. J. Wenk M. R. Yao S. Q. (2010). Activity-based proteome profiling of potential cellular targets of Orlistat--an FDA-approved drug with anti-tumor activities. J. Am. Chem. Soc. 132 (2), 656–666. 10.1021/ja907716f 20028024

[B88] Yanovski S. Z. Yanovski J. A. (2014). Long-term drug treatment for obesity: a systematic and clinical review. JAMA 311 (1), 74–86. 10.1001/jama.2013.281361 24231879 PMC3928674

[B89] Yin F. Yang J. Luo H. Yu T. Lu W. Zhao M. (2025). Targeting oncogenic activation of FLT3/SREBP/FASN promotes the therapeutic effect of quizartinib involving disruption of mitochondrial phospholipids. Cell Death Dis. 16 (1), 327. 10.1038/s41419-025-07661-6 40263296 PMC12015539

[B90] Zelber-Sagi S. Kessler A. Brazowsky E. Webb M. Lurie Y. Santo M. (2006). A double-blind randomized placebo-controlled trial of orlistat for the treatment of nonalcoholic fatty liver disease. Clin. Gastroenterol. Hepatol. 4 (5), 639–644. 10.1016/j.cgh.2006.02.004 16630771

[B91] Zhao S. Mugabo Y. Iglesias J. Xie L. Delghingaro-Augusto V. Lussier R. (2014). alpha/beta-hydrolase domain-6-accessible monoacylglycerol controls glucose-stimulated insulin secretion. Cell Metab. 19 (6), 993–1007. 10.1016/j.cmet.2014.04.003 24814481

[B92] Zhou Z. Niu Y. Ma Y. Zhang D. Wang Y. Ji R. (2025). Lipid accumulation inhibition strategies alleviate fusobacterium nucleatum-infected colorectal cancer. Microbiome 13 (1), 181. 10.1186/s40168-025-02133-7 40770383 PMC12326762

